# New mechanistic insights into hepatoprotective activity of milk thistle and chicory quantified extract: The role of hepatic Farnesoid-X activated receptors

**DOI:** 10.22038/AJP.2020.17281

**Published:** 2021

**Authors:** Azadeh Khalili, Parviz Fallah, Seyed Ali Hashemi, Mohammad Mahdi Ahmadian-Attari, Vahid Jamshidi, Roham Mazloom, Leila Beikzadeh, Gholamreza Bayat

**Affiliations:** 1 *Department of Physiology-Pharmacology-Medical Physic, School of Medicine, Alborz University of Medical Sciences, Karaj, Iran*; 2 *Evidence-based Phytotherapy and Complementary Medicine Research Center, Alborz University of Medical Sciences, Karaj, Iran *; 3 *Department of Medical Laboratory Sciences, Faculty of Para-Medicine, Alborz University of Medical Sciences, Karaj, Iran*; 4 *Department of Pathology, School of Medicine, Alborz University of Medical Sciences, Karaj, Iran*; 5 *Department of Pharmacognosy, Faculty of Pharmacy, Alborz University of Medical Sciences, Karaj, Iran*; 6 *Department of Pharmacology and Toxicology, Faculty of Pharmacy, Pharmaceutical Sciences Branch, Islamic Azad University, Tehran, Iran*

**Keywords:** Farnesoid X receptors (FXR), Acetaminophen, Milk thistle, Chicory, Necrosis

## Abstract

**Objective:**

Farnesoid-X-activated receptors (FXR) are key modulators of liver regeneration. Milk thistle and Chicory are known as potent protective remedies in several liver disorders. The objective of this work was to examine the role of *FXR* in the hepato-healing properties of milk thistle (MTE) and chicory extracts (CE) in a rat model of acetaminophen-induced hepatotoxicity.

**Materials and Methods:**

Male Wistar rats were randomly divided into seven groups including control, vehicle, acetaminophen (500 mg/kg/day, oral), acetaminophen plus oral MTE 200 and 400 mg/kg/day, and acetaminophen plus oral CE 500 and 1000 /kg/day for 28 days. Liver function and histology as well as the pattern of hepatic *FXR* expression were assessed after 4 weeks.

**Results:**

Administration of acetaminophen was associated with a significant elevation of liver transaminase along with the architectural injuries. In contrast, chronic concomitant administration of both MTE and CE significantly restored the liver function and structural abnormality. The main molecular findings of the study revealed that the lower doses of both MTE and CE led to a marked upregulation of hepatic *FXR* expression.

**Conclusion:**

Discovery of the involvement of the nuclear modulating pathways in hepatoprotective activity of the extracts, providesa new mechanistic insight which needs further investigations.

## Introduction

Several kinds of chemicals and diseases may threaten the liver which depends on the severity of the effect on the hepatic architecture and/or function. In spite of dramatic advances in drug development technology, there is no specific approved drug for chronic and intensive liver damages. It is probably because liver regeneration is not easily possible in advanced progressive failure stages. Some pathological pathways such as oxidative stress and inflammation have a pivotal role in the development and progression of the liver diseases (Jassim, 2013 ▶; Del Campo et al., 2018 ▶). Although the exact mechanistic pathways of liver diseases are not fully understood, several molecular mechanisms have been documented. Farnesoid-X activated receptor, known as *FXR*, is one of the metabolic nuclear receptors with high hepatic expression which is a master regulator of the bile acid synthesis, conjugation, and enterohepatic circulation (Pathak et al., 2017 ▶), as well as lipid and glucose metabolism (Jiang et al., 2015 ▶; Taoka et al., 2016 ▶). Nowadays, this ligand-gated nuclear receptorsis also considered a key regulator in liver regeneration (Li and Guo, 2015 ▶). It is endogenously activated by bile acids particularly chenodeoxycholic acid (CDCA) (Akhondzadeh et al., 2005 ▶). Several clinical and experimental studies indicated that dysregulation of the hepatic *FXR* gene expression and/or activity is strongly correlated with the development of chronic liver damages (Zhang et al., 2009 ▶; Lee et al., 2010 ▶).

According to the long-time traditional medicine experiences and experimental and clinical studies, some medicinal herbs are considered potent liver protecting agents. Two medicinal herbs of the Asteraceae family, including milk thistles (*Silybum marianum*) and chicory (*Cichorium intybus*), are pharmacologically effective in prevention and even treatment of liver diseases (Kailash and Swatantra Kumar, 2016 ▶; Sadat Sharifi and Bakhshaei, 2017 ▶). Based on this documented hepatoprotective activity and the protective role of FXR in several liver diseases, the present study was designed to find out the possible role of nuclear receptors of FXR in the hepato-healing properties of the two herbs. 

## Materials and Methods

**Chemicals**

Acetaminophen powder was obtained from DarouPakhsh Pharmaceutical Manufacturing Company (Temad Co., Karaj, Iran). Ketamine and Xylazine were purchased from Alfasan (Woerden, Holland). Chicory root (*C.intybus* L.) and milk thistle seed (*S.marianum* L.) were purchased from Tehran botanical market and authenticated by the Herbarium of the School of Traditional Medicine, ShahidBeheshti University of Medical Sciences (voucher No. *HMS-516* and *HMS-517* for *S.marianum* and *C.intybus*, respectively). 

**Preparation of the extracts**

**Chicory extract preparation**

To prepare the aqueous extract of chicory root, after washing the roots with cold water, we left them to dry in air at room temperature. Then, the roots were crushed and extracted by 60°C water via percolation method (water/dry root ratio 8:1; extraction time: 10 hr). The extract was filtered and dried using aspray dryer. Feed flow rate was 20 l/hr with inlet and outlet air temperature of 190±2 and 75±2°C, respectively.

**Milk thistle extract preparation**

The methanolic extract of milk thistle seeds was prepared by percolation method using 99.99% (v/v) methanol. The extract was concentrated using avacuum rotary evaporator (Heidolph, Germany) and left to dry in a desiccator. The extraction yield (w/w) of both herbal extracts was calculated as the weight of dry extract/weight of dry starting material×100.

**Quantification of active ingredients in the extracts**

 The main active constituents of the aqueous extract of *C.intybus* (as inulin) and methanolic extract of *S.marianum* (Silymarin: as silibinin) was determined by the quantitative high-performance liquid chromatographic (HPLC: Knauer, Germany) and UV-spectrophotometry (Spectro UV-VIS double beam pc scanning spectrophotometer UVD 2960) methods, respectively.

**Animals**

Eight-week-old male Wistar rats, weighing 200–250g, were obtained from Royan Animal Breeding Center, Karaj, Iran. They were kept under standard conditions (12 hr light/dark cycle at 20–24°C and 50±5% relative humidity). Animals had free access to food and water during the study. The animal care and experimentation were performed according to the national guidelines and protocols approved by the Research Ethics Committee of Alborz University of Medical Sciences in accordance with the National Institute of Health Guide for the Care and Use of Laboratory Animals (NIH Publication No.85-23, revised 1996). 

**Experimental design and protocol**

Forty-nine animals were randomly divided into 7 groups (n=7 rats in each) including a control group, a vehicle group assigned to received 0.3% Carboxy methyl cellulose (CMC)as vehicle, an acetaminophen group assigned to receive acetaminophen at 500 mg/kg/day (oral, suspended in 0.3% CMC ), two groups assigned to receive acetaminophen (500 mg/kg/day, oral) concomitant with chicory extract (CE; 500 and 1000 mg/kg/day,oral), and two groups assigned to receive acetaminophen (500 mg/kg/day, oral) concomitant with Milk Thistle extract (MTE, suspended in 0.3% CMC at 200 and 400 mg/kg/day, oral).

During the study period (28 days), all solutions were prepared freshly just prior to daily administration and given once a day at the same time. In all extract-treated groups, animals received the assigned dose 1 hrafter administration of acetaminophen. For detection of any sign of morbidity and/or mortality, the animals were observed twice a day. At the end of the study, under deep surgical anesthesia using intraperitoneal injections of ketamine (60 mg/kg) and xylazine (8 mg/kg), bilateral thoracotomy was performed, and blood samples were obtained gently from the right ventricle. 

**Determination of Serum biochemical parameters**

Blood samples were collected to determine the serum levels of some biochemical markers including albumin (ALB), aspartate aminotransferase (AST), alanine aminotransferase (ALT), alkaline phosphatase (ALP) lactate dehydrogenase (LDH) Gamma-glutamyltransferase (GGT), and total and direct bilirubin using Pars Azmun commercial kits (Pars Azmun Co, INC, Karaj, Iran), according to the manufacturer’s guidelines.

**Histopathological assessments**

For histopathological assessments, the largest right lobe of each liver was removed and immediately fixed in a 10% formalin solution. After dehydration and clearance, the samples were embedded in paraffin wax and sectioned into a 5-μm thickness. Tissue staining using Hematoxylin and Eosin (H&E), Masson's Trichrome, and Reticulin was performed for detecting any pathological signs of toxicity, fibrotic scars, or necrotic lesion, respectively.

**Hepatic** ***FXR*** **gene expression using real time RT-PCR technique**

 Preparation of the samples to identify the expression of the hepatic *FXR* was done according to the protocol (Safari et al., 2014 ▶). Briefly, about 50 mg of the hepatic tissue was homogenized using a polytron tissue homogenizer (DAIHAN-brand Homogenizing Stirrer, HS-30E; Korea). RNA was then extracted using Trizol (Qiagen) based on the manufacturer’s instructions. Then, the cDNA synthesis was performed using Reverse Transcriptase cDNA synthesis kit (Fermentas), based on the protocol. Expression of *FXR *was measured by Real-Time PCR using SYBR GREEN (TAKARA). The experiments were performed in duplicates as follows: denaturation at 95°C for 10 min followed by 45 cycles at 95°C for 10 sec and 60°C for 10 sec and 72°C for 10 sec. The expression level of *FXR *was normalized to 

that of *GAPDH *gene and expressed as fold-change ratio. The exact nucleotide sequences of the *FXR *and* GAPDH *primers are shown in Table 1. 

**Statistical analysis**

Data presented as Mean±SEM were analyzed using One-way analysis of variance (ANOVA) followed by Duncan's multiple range test for between groups comparisons. A *P*-value of <0.05 was considered statistically significant. The quantification of gene expressions was analyzed and plotted using REST 2009 (Technical University Munich, Germany) and GraphPad Prism 8.0.2 GraphPad Software Inc., San Diego, CA, USA) software, respectively. 

## Results

**Analytical assessment of MTE and CE**

**Figure 1:**
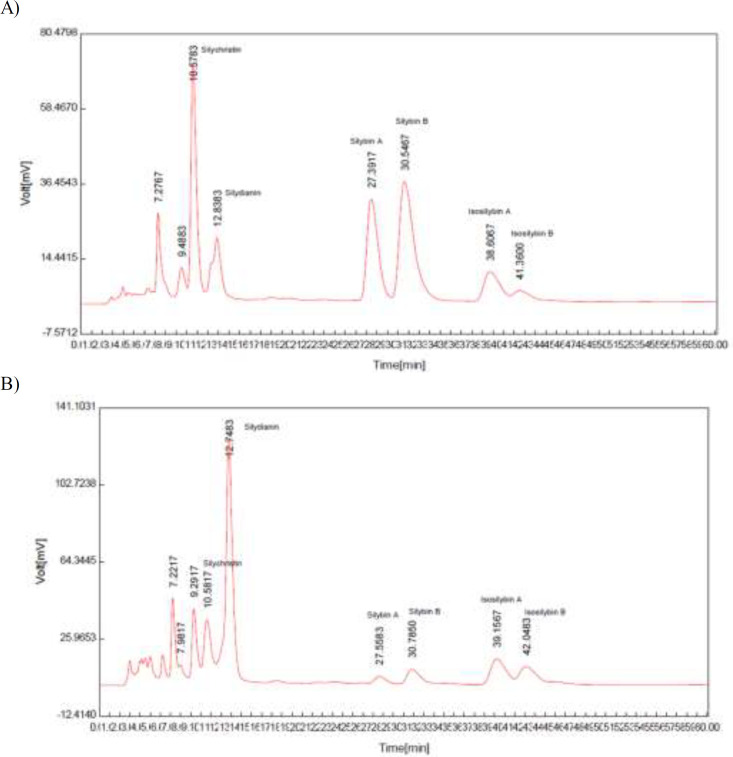
Typical HPLC Chromatograms of A) standard silymarin and B) methanolic extract of Milk Thistle seeds (as sample). HPLC instrument (Knauer, Germany) equipped with an Agilent Knauer- UV K2501diode array detector, Knauer- K1001 pump, Agilent Eclipse-XDB-C18 analytical column (125 mm, 4.6 mm, 5μm). The aqueous mobile phase A: phosphoric acid R, methanol R, water R (0.5:35:65 V/V/V) mobile phase B: phosphoric acid R, methanol R, water R (0.5:50:50 V/V/V), mobile phase flow rate 0.8 ml/min, injection volume 20μl

The HPLC chromatograms of standard silymarin and MTE sample are shown in Figure 1. The total amount of active ingredients as silibinin was 50.2% (W/W%) in the MTE sample. Moreover, spectrophotometric UV-Vis analysis of CE revealed that the inulin content of the CE was 43.58% (W/W%).

**Histopathological examination**

**Histopathological changes in the liver following acetaminophen treatment**

**Figure 2:**
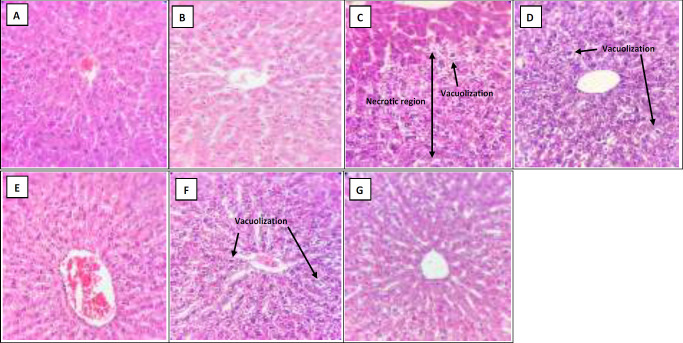
Hepatic Hematoxylin-Eosin (H&E)-stained sections (X400) in the (A) Control, (B) Vehicle, (C) Acetaminophen, (D and E) Milk Thistle extract (MTE 200 and 400 mg/kg/day) and (F and G) Chicory extract (CE 500 and 1000 mg/kg/day) groups after 28 days

**Figure 3:**
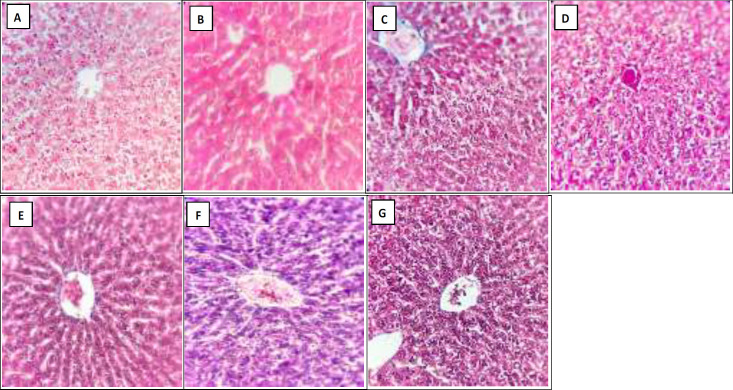
Hepatic Masson’s trichrome stained sections (X400) in the (A) Control, (B) Vehicle, (C) Acetaminophen, (D and E) Milk Thistle extract (MTE 200 and 400 mg/kg/day) and (F and G) Chicory extract (CE 500 and 1000 mg/kg/day) groups after 28 days

**Figure 4:**
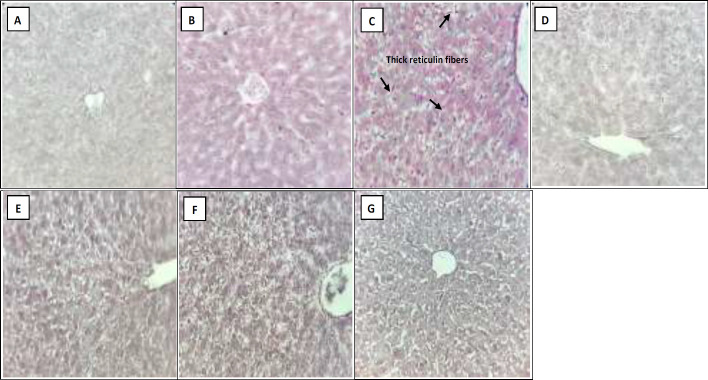
Hepatic Reticulin stained sections (X400) in the (A) Control, (B) Vehicle, (C) Acetaminophen, (D and E) Milk Thistle extract (MTE 200 and 400 mg/kg/day) and (F and G) Chicory extract (CE 500 and 1000 mg/kg/day) groups after 28 days

As H&E (Figure 2), Masson's trichrome (Figure 3) and reticulin (Figure 4) staining showed, administration of vehicle did not show any sign of histological alteration compared to the control group. Chronic administration of acetaminophen, however, led to remarkable hepatotoxicity which was characterized by significant congestion, sinusoidal dilation, vacuolization, and necrosis. With lower degrees, other signs of liver toxicity such as inflammatory infiltration, Kupffercell hyperplasia, bile stasis and plugs as well as pyknosis were seen. Although Masson's trichrome staining did not detect any signs of fibrotic bundles following acetaminophen treatment (Figure 3), a dense network of thick reticulin fibers was developed in acetaminophen-treated group (Table 1and Figure 4).

**Histopathological changes in the liver following CE treatment**

According to H&E staining (Figure 2 and Table 1), co-administration of CE with acetaminophen, significantly reversed the signs of acetaminophen-induced liver injury. The observed hepatoprotective effects of both 500 and 1000 mg/kg/day of CE were relatively similar (Table 1). Moreover, thick reticulin fibers markedly disappeared due to CE administration (500 and 1000 mg/kg/kg) (Figure 4).

**Histopathological changes in the liver following MTE treatment**

As shown in Figure 2 and Table 1, concomitant administration of MTE and acetaminophen was also accompanied bymarked improvement of histopathological injuries induced by chronic administration of acetaminophen.

**Table 1:** The exact nucleotide sequences of the *FXR *and* GAPDH *primers

Genes	Forward	Reverse
*FXR*	TGGGAATGTTGGCTGAATG	CCTGTGGCATTCTCTGTTTG
*GAPDH*	GCCTTCTCTTGTGACAAAGTG	CTTCCCATTCTCAGCCTTG

There was no significant difference in the healing properties of the two doses of MTE (200 and 400 mg/kg/day). In addition, administration of MTE at both doses markedly removed the thick reticulin fibers (Figure 4).

**Serum biomarker assessment results**

**The effects of chronic administration of vehicle or acetaminophen**

Compared to the control group, administration of the vehicle was not associated with remarkable changes in the serum levels of ALT, AST, GGT, LDH, ALP and ALB. However, chronic administration of acetaminophen led to a significant increase in ALT (p<0.05), AST (p<0.01) and LDH (p<0.01), but not GGT, ALP, ALB, or total and direct bilirubin serum levels (Table 2).

**The effects of chronic administration of CE (500 and 1000 mg/kg/day)**

As shown in Table 2, chronic administration of CE500 was associated with a significant reduction in ALT (p*<*0.001), AST (p*<*0.001), LDH (p*<*0.01) and ALP (p*<*0.001) serum levels compared to the acetaminophen-treated group. The LDH decline was obviously more pronounced at the dose of 1000 mg/kg/day compared to 500mg/kg (p*<*0.05). In contrast, the ALP reduction at 500 mg/kg/day was more significant compared to 1000 mg/kg/day (p*<*0.05). The serum levels of GGT, ALB, total and direct bilirubin did not show a significant change among the experimental groups.

**The effects of chronic administration of MTE (200 and 400 mg/kg/day)**

According to Table 2 and compared to the acetaminophen-treated group, serum levels of ALT (p<0.01), AST (p<0.001) and ALP (p<0.01) were significantly reduced in the MTE200 and 400 groups. Moreover, compared to the acetaminophen-treated group, administration of MTE400 was associated with a significant LDH level reduction (p<0.05). The serum levels of GGT, ALB, and total and direct bilirubin did not show significant changes among the experimental groups. 

**Real time RT-PCR gene expression results**

**Alteration in the hepatic** ***FXR*** **mRNA expression due to vehicle or acetaminophen treatment**

**Figure 5:**
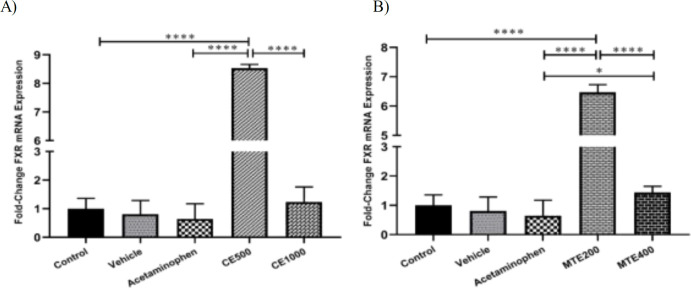
Effect of chronic administration of acetaminophen, chicory (A) and milk thistle (B) extracts on the expression of hepatic *FXR*gene. Experimental groups including Control, Vehicle (0.3% CMC), Acetaminophen (500 mg/kg/day, oral), Milk Thistle extract 200 mg/kg/day, oral (MTE200), 400 mg/kg/day, oral (MTE400), Chicory extract 500 mg/kg/day, oral (CE500) and 1000 mg/kg/day, oral (CE1000). The duration of the study was 4 weeks. Data are presented as mean±SEM (n=7 in each). *p<0.05 and****p<0.0001

The findings of the real time RT-PCR method revealed that, compared to the control group, chronic administration of the vehicle was not associated with significant changes in the expression of hepatic *FXR* gene. Hepatic expression of *FXR* in the acetaminophen-treated group was also accompanied bya non-statistically significant reduction in comparison to the control one (Figure 5). 

**Alteration in the hepatic** ***FXR*** **mRNA expression due to CE treatment**

The hepatic expression of *FXR* showed a dose-reversal pattern (Figure 5A). In comparison to the higher dose, administration of the lower dose of CE significantly increased the expression of hepatic *FXR*; the observed up-regulation of the *FXR* following 500 mg/kg/day of CE was 8.53 (p*<*0.0001), 13.22 (p*<*0.0001) and 6.85 (p*<*0.001) folds when compared to the control, acetaminophen alone and the high dose of CE, respectively.

**Table 2:** Histological characteristics of H&E and Trichrome stained liver sections in the control, vehicle-treated, acetaminophen-treated (500 mg/kg/day), Milk Thistle Extract treated (MTE 200 and 400 mg/kg/day) and chicory extract treated groups (CE500 and 1000 mg/kg/day) groups after 28 days

	**Groups**	**Control**	**Vehicle**	**Acet.500**	**MTE200**	**MTE400**	**CE500**	**CE1000**
**Number**	**H&Estaining**							
**1**	Glycogen depletion	0	0	+	+	0	0	0
**2**	Hemorrhage	0	0	0	0	0	0	0
**3**	Congestion	0	0	+++	++	++	++	++
**4**	Sinusoidal dilation	0	0	++	+	+	+	+
**5**	Edema	0	0	++	0	+	+	+
**6**	Inflammatory infiltration (Lymphocytic infiltration)	0	0	+	+	+	+	0
**7**	Vacuolization	0	0	++++	+++	++	++	+
**8**	Bile stasis	0	0	+	+	0	0	0
**9**	Bile plugs	0	0	+	+	0	0	0
**10**	Kupffer cell hyperplasia	0	0	+	+	+	+	++
**11**	Pyknosis	0	0	+	+	0	0	0
**12**	Necrosis	0	0	++	++	0	0	0
**Masson's Trichrome staining**								
**Fibrosis**		0	0	0	0	0	0	0
**Reticulin staining**								
**Necrosis **		0	0	++	0	0	0	0

Histological changes were scored as none (0), active damage less than 25% (+), active damage less than 50% (++), active damage less than 75% (+++) damage and active damage more than 75% (++++).

Histological changes were scored as none (0), active damage less than 25% (+), active damage less than 50% (++), active damage less than 75% (+++) damage and active damage more than 75% (++++).

**Table 3:** The levels (mean±SEM, n=7) of serum biochemical markers including aspartate aminotransferase (AST), alanine aminotransferase (ALT), lactate dehydrogenase (LDH) and gamma-glutamyltransferase (GGT), alkaline phosphatase (ALP), albumin (ALB), total (Total Bil) and direct bilirubin (Direct Bil) in the control, vehicle-treated, acetaminophen-treated (500 mg/kg/day), Milk Thistle Extract treated (MTE 200 and 400 mg/kg/day) and chicory Extract treated (CE500 and 1000 mg/kg/day) groups after 28 days

	**AST**	**ALT**	**LDH**	**GGT**	**ALP**	**Alb**	**Total Bil**	**Direct Bil**
**Control**	104.0±1.51	70.0±2.50	695.0±76.88	2.5±0.64	837.6±48	3.78±0.07	0.20±0.03	0.10±0.00
**Vehicle**	110.3±3.60	73.16±2.15	759.8±39.88	3.7±0.54	767.5±32.2	3.68±0.08	0.20±0.00	0.13±0.01
**Acet.**	169.0±15.67**	148.2±13.76*	1119.4±136.8**	2.71±0.96	850.0±56.52	3.64±0.06	0.25±0.02	0.11±0.01
**MTE200**	104.5±6.6**††**	65.7±6.0**†††**	898.0±58.3*	2.5±0.5	636.3±45††*	3.65±0.11	0.22±0.03	0.10±0.01
**MTE400**	118.0±6.5**††**	72.5±5.5**†††**	787.5±47.8**†**	3.5±0.95	640.2±43.3††*	3.68±0.05	0.24±0.02	0.11±0.03
**CE500**	92.2±2.5**†††**	63.5±7.1**†††**	733.6±43.8**††**	2.2±0.47	616.1±46.4**††	3.80±0.05	0.22±0.02	0.12±0.02
**CE1000**	98.8±3.9**††**	65.0±2.00**†††**	594.2±41.6**††●**	3.0±0.22	803.5±23.7**●**	3.82±0.05	0.21±0.03	0.10±.02

*Significant difference compared tothe control group (*p<0.05 and**p<0.01); †significant difference compared tothe acetaminophen-treated group (†p<0.05, ††p<0.01 and †††p<0.001); ●significant difference compared to the CE500-treated group (●p<0.05)

In contrast, administration of 1000 mg/kg/day of CE, was not accompanied bysignificant alterations in the gene expression in comparison to the control or acetaminophen groups (1.24 vs. 1). As a clear finding, in comparison to lower dose of CE, the higher dose of CE significantly reduced the relative gene expression of the hepatic *FXR *(1 vs. 0.14, p*<*0.0001). 

**Alteration in the hepatic** ***FXR*** **mRNA expression due to MTE treatment**

As shown in Figure 5B, the expression level of hepatic *FXR *gene was negatively correlated with the administered dosage of MTE. Compared to the controls, chronic administration of 200mg/kg/day led to a 6.48-fold (p*<*0.001) increase in the *FXR *mRNA expression. Moreover, the expression of *FXR* was 10.04 (p*<*0.0001) and 4.48 (p*<*0.0001) times higher in the 200 mg/kg/day group compared to the acetaminophen alone and 400 mg/kg/day groups, respectively. There was no change in the expression of *FXR* following administration of MTE at 400mg/kg/day although a2.24-fold up-regulation was seen compared to the acetaminophen-treated group (p*<*0.04). Generally, compared to the lower dose of MTE, administration of the higher dose was accompanied by a significant *FXR* mRNA down-regulation (0.22 vs. 1, p*<*0.0001).

## Discussion

The present study was designed to investigate the role of Farnesoid-X-activated receptors in the hepatoprotective effect of Milk Thistle and Chicory extracts. As the main findings of the experiment, different doses of the extracts exhibited a different *FXR* expression pattern; lower doses in either MTE or CE were associated with marked up-regulation in hepatic *FXR* gene expression, whereas the dose increment in both groups led to considerable down-regulation of the gene. Indeed, a negative correlation was observed between the *FXR* gene expression and the level of the dose. Biochemical and histological findings were also similar to those of other previous studies and confirmed the hepatoprotective roles of the extracts for both administered doses. In the present study, however, the chronic administration of acetaminophen was not associated with marked reductions in the expression of hepatic *FXR* mRNA level.

Acetaminophen-induced hepatic injury is one the practical experimental models of hepatotoxicity characterized by serum abnormality along with histopathological deformity. In line with previous reports (Adil et al., 2016 ▶; Mazraati and Minaiyan, 2018 ▶), administration of acetaminophen in the present study was associated with marked alteration ofthe serum levels of liver enzymes besides obvious histopathological injury. In contrast to our data, Adil et al. findings showed that administration of acetaminophen at 700 mg/kg/day for 14 days was accompanied with a significant down-regulation of hepatic *FXR *(Adil et al., 2016 ▶). Although the studies were not completely conducted underthe same conditions, it seems that the effect of acetaminophen on the expression of hepatic *FXR* is more dose-dependent than time-dependent.

Acute and chronic liver diseases are one of leading causes of mortality and morbidity all around the world. The etiology and pathophysiology of these major disorders are not fully known, but there is emerging evidence which uncovers some underlying molecular mechanisms involved in the disease state, in which the role of some specific nuclear receptors, such as farnesoid receptor, is identified. The *FXR* physiologically plays a major role in modulating the bile acid synthesis and hemostasis in the body (Li and Chiang, 2013 ▶; Jacinto and Fang, 2014 ▶). In addition, it has an important role in glucose and lipid regulatory pathways (Li and Guo, 2015 ▶; Hylemon et al., 2017 ▶). Several clinical and experimental studies support the concept that there is a negative correlation between the hepatic *FXR* gene expression and development or worsening of the liver disease state (Zhang et al., 2009 ▶; Lee et al., 2010 ▶). As Adil et al. showed, acetaminophen-induced hepatotoxicity was associated with marked down-regulation of hepatic *FXR* mRNA level (Adil et al., 2016 ▶). Moreover, decrease in bile acid receptor expression in experimental models of liver fibrosis, is another evidence which indicates the crucial role of *FXR* in the liver pathology. According to Verbeke et al. findings, using a potent selective FXR agonist, obeticholic acid (INT-747), in two models of cirrhotic rats, including bile duct ligation (BDL) and thioacetamide-induced toxicity, led to marked improvement ofendothelial vasodilation via activation of intrahepatic eNOS (endothelial-derived nitric oxide synthase) pathway (Verbeke et al., 2014 ▶). In addition, some experimental models of *FXR*-deficient animals also confirmeda protective role of these types of nuclear receptors in the liver function and/or architecture (Su et al., 2012 ▶; Kong et al., 2016 ▶). In contrast, there is some evidence showing a significant up-regulation of hepatic *FXR* expression in some liver disorders (Aguilar-Olivos et al., 2015 ▶). There are also several reports indicating the anti-inflammatory and anti-fibrotic roles of *FXR* activation in experimental animal models (Shaik et al., 2014 ▶; Massafra et al., 2016 ▶). As Verbeke et al. showed, 4 week administration of obeticholic acid in thioacetamide-induced cirrhotic rats accompanied by marked reduction of pro-inflammatory cytokines and pro-fibrotic markers during thioacetamide-administration, strongly reversed the established cirrhosis (Verbeke et al., 2016 ▶). Despite the above points, there are some discrepancies regarding the protective role of *FXR* up-regulation in hepatic disorders. A more recent study revealed that using obeticholic acid in reversible bile duct ligated rats for 7 days, was associated with biliary injury exacerbation which was secondary due to up-regulation of the bile salt export pump (van Golen et al., 2018 ▶). 

Several species of medicinal plants have historically been considered therapeutic targets in the prevention, palliation and/or treatment of liver disease signs and symptoms. In this regard, two medicinal herbs from the Asteraceae family, including milk thistle (*S.marianum*) and chicory (*C.intybus*), are well-known for their hepato-healing properties. Milk Thistle standard extract obtained from seeds of *S.marianum*, known as silymarin, is composed of 7 flavonolignans and polyphenols, in which, silibinin is considered the main active ingredient (Bijak, 2017 ▶). There are several clinical and experimental investigations which confirm the hepatoprotective role of the extracts and/or their active components. A marked reduction in the plasma levels of liver enzymes such as ALT, AST and ALP by silymarin (de Avelar et al., 2017 ▶) has been frequently reported. Moreover, administration of silymarin in liver disease with different etiologies, led to significant restoration of histopathological and structural abnormality of the liver (Surai, 2015 ▶; de Avelar et al., 2017 ▶). Despite extensive studies, the exact mechanism of the action of silymarin and/or its active component is not fully understood. In this regard, considerable attention has been paid to its anti-oxidative (Stiuso et al., 2014 ▶) and anti-inflammatory properties (Verbeke et al., 2016 ▶; Tsaroucha et al., 2018 ▶). This appears to occur in different ways including direct free radical scavenging activity, inhibition of reactive radical species formation, and mitochondrial function restoration (Surai, 2015 ▶). In addition, according to other related studies, the potent anti-inflammatory property of the extract is due to itsinhibitory effect on the main transcriptional factor of NF-κB (Stevenson and Hurst, 2007 ▶; Gupta et al., 2014 ▶; Surai, 2015 ▶). The latter is involved in several key processes such as inflammatory response, cell differentiation, and apoptosis (Surai, 2015 ▶). Recently, a valuable literature survey has shown that the *FXR* plays a key modulator role in several metabolic and inflammatory processes (Shaik et al., 2015 ▶). The hepatoprotective action of chicory extract in the literature is also attributed to its prominent antioxidant (Li et al., 2014 ▶; Soliman et al., 2016 ▶) and anti-inflammatory activity (Cavin et al., 2005 ▶). 

Given the protective role of *FXR* which was documented in several lines of evidence, the present study focused on the role of this type of nuclear receptors in the pharmacological effects of MTE and CE. Although biochemical or histopathological findings did not show any negative dose-response relationship, the observed reverse correlation between the dose (for both MTE and CE) and hepatic *FXR* expression was a considerable point. As compared to the lower doses, the higher ones were surprisingly associated with considerable down-regulation of hepatic *FXR*. Recently, Adil and his colleagues evaluated the protective effects of naringin against acetaminophen-induced hepatic and renal toxicity (Adil et al., 2016 ▶). They showed that naringin pretreatment markedly restored the hepatic *FXR* mRNA expression which was damaged by chronic administration of acetaminophen. The study also used silymarin as the positive control at a single daily dose (25 mg/kg/day for 2 weeks), and at the administered dose, it corrected the mRNA expression of the hepatic *FXR*(Adil et al., 2016 ▶). According to the present findings, it is not clear why administration of higher doses of MTE or CE was associated with obvious reduction ofthe hepatic expression of *FXR*. The observed down-regulation was the same as the control ones and was not a pathological down-regulation. Interestingly, toxicological findings of chicory showed that compared to the lower dose, higher doses of chicory extract increased the CCL_4_-induced cytotoxicity inisolated hepatocytes (Jamshidzadeh et al., 2010 ▶). They also showed that chicory extract at higher doses did not protect the liver against CCL_4_-induced hepatotoxicity (Jamshidzadeh et al., 2010 ▶). In spite of the present findings, there is some evidence indicating that toxicological evaluation of chicory root extract did not show any sign of obvious toxicity in both chronic and acute toxicological assessment tests (Schmidt et al., 2007 ▶; Conforti et al., 2008 ▶). Therefore, such a dose-independent expression pattern of hepatic *FXR* might be due to participation of other regulatory signaling pathways.

The main limitation of this study was lack of information on alteration of glutathione modulating pathway and its relationship with the *FXR* gene expression. Further specific molecular investigations are recommended to be conducted to elucidate the exact mechanism(s) of the observed dose-reversal relationship and answer the question whether they are foe or friend at higher doses.
